# Correlation between right ventricular–pulmonary artery coupling and the prognosis of patients with pulmonary arterial hypertension

**DOI:** 10.1097/MD.0000000000017369

**Published:** 2019-10-04

**Authors:** Lin Nie, Jun Li, Sanping Zhang, Yaling Dong, Ming Xu, Menghuan Yan, Gangcheng Zhang, Laichun Song

**Affiliations:** a Department of Cardiac Surgery; b Department of Anaesthesiology, Wuhan Asia Heart Hospital, Wuhan; c Department of Cardiology, Third People's Hospital of Xining, Qinghai; d Department of Cardiology, Wuhan Asia Heart Hospital, Wuhan, P.R. China.

**Keywords:** pulmonary arterial hypertension, right ventricular–pulmonary artery coupling, survival

## Abstract

This study aimed to analyze the correlation between the efficiency coefficient of right ventricular–pulmonary artery coupling (*η*_vv_) and the prognosis of patients with pulmonary arterial hypertension (PAH).

A total of 64 patients who underwent right heart catheterization (RHC) were enrolled and divided into PAH and control groups depending on the RHC results. Pressure and volumetric methods were adopted to analyze the results of RHC and cardiac magnetic resonance imaging examination. The *η*_vv_ of patients in 2 groups were calculated, and the relationship between *η*_vv_ calculated by the 2 methods and the 2-year prognosis of patients with PAH was evaluated.

The hemodynamic index and right ventricular–pulmonary artery coupling parameter of patients with PAH were significantly higher than those in the control group (*P* < .05). The right ventricular volume parameter in the PAH group was significantly different from that in the control group (*P* < .05). For patients with PAH, the end-systolic elastance/effective arterial elastance (*E*es/*E*a) calculated by the volumetric method was significantly related to the prognosis of patients (odds ratio = 0.192, 95% confidence interval: 0.042–0.868, *P* = .032). When *E*es/*E*a <0.67 was calculated by the volumetric method, the adverse prognosis of patients with PAH increased significantly (*P* < .05).

The *E*es/*E*a calculated by the volumetric method may be better an independent factor for the prognosis of patients with PAH.

## 1 Introduction

Pulmonary arterial hypertension (PAH) is a complex and comprehensive disease, which may cause multiple-organ dysfunction and have a poor prognosis. Therefore, it is regarded as cancer in the field of pulmonary vascular disease. Among many factors related to the prognosis of patients with PAH, the right ventricular function is one of the undoubtedly important reasons. In recent years, some scholars have proposed the concept of “ventricular–vascular coupling” considering the matching of right ventricular function and its circulation.^[1]^ In the physiological state, ventricular and vascular elastance could be dynamically adjusted to maintain the optimal coupling relationship, but under the pathological conditions, ventricular and vascular elastance changed and could not achieve the optimal coupling relationship. Clinical studies also confirmed the optimal coupling relationship ensuring that most of the energy pumped from the ventricle was transmitted to blood vessels.^[2]^ Scholars proposed the concept of coupling efficiency coefficient (*η*_vv_) to further quantify the relationship of ventricular and vascular interaction with optimal coupling.^[3]^ The quantitative evaluation of right heart function was performed by calculating the ratio of ventricular elastance (end-systolic elastance [*E*es]) and vascular elastance (effective arterial elastance [*E*a]), that is, *η*_vv_ = *E*es/*E*a. The aim of this study was to measure the efficiency coefficient of right ventricular–pulmonary artery coupling (*η*_vv_) in patients with PAH using different methods based on the data obtained from right cardiac catheterization and cardiac magnetic resonance imaging (MRI) examination. It was also performed to explore the significance of right ventricular–pulmonary coupling predicting the long-term prognosis of patients with PAH by evaluating the right ventricular function of patients and comparing its correlations with hemodynamic parameters and right ventricular volume, thereby providing a basis for finding a simple, practical, and accurate clinical index to reflect the changes in condition and the prognosis of patients.

## 2 Patients and methods

### 2.1 Patients

This study was approved by the Institutional Review Board of Wuhan Asia Heart Hospital and was in compliance with the Health Insurance Portability and Accountability Act regulations and the Declaration of Helsinki. The Institutional Review Board waived the need for individual patient consent. According to the guidelines for PAH^[4]^ issued by the European Society of Cardiology in 2015, right cardiac catheterization was performed in all patients with suspected PAH (aged ≥18 years) at Wuhan Asia Heart Hospital in January 2015. The hemodynamic parameters were recorded, including mean pulmonary artery pressure (mPAP), right ventricular end-systolic pressure (RVESP), right ventricular end-diastolic pressure (RVEDP), pulmonary vascular resistance (PVR), and cardiac output (CO). Under the resting state, if mPAP ≥25 mm Hg and PVR ≥3 Wood units, it could be diagnosed as PAH. Patients with absolute contraindications for cardiac MRI examination and those with severe systemic disease were excluded. Cardiac MRI was performed within 48 hours after right heart catheterization (RHC). Also, right ventricular volume parameters were recorded, including right ventricular end-systolic volume, right ventricular end-diastolic volume, stroke volume (SV), and right ventricular ejection fraction. All the patients underwent normative and regular targeted PAH therapy, including endothelin receptor antagonists and phosphodiesterase inhibitor. The clinical endpoint was defined as all-cause death and clinical deterioration. Clinical deterioration included worsening of the patients’ condition. The patients were admitted to the hospital again, their condition worsened, and interventions such as medication adjustment and lung transplantation were needed.

### 2.2 Calculation of right ventricular–pulmonary artery coupling efficiency

**Figure 1:**
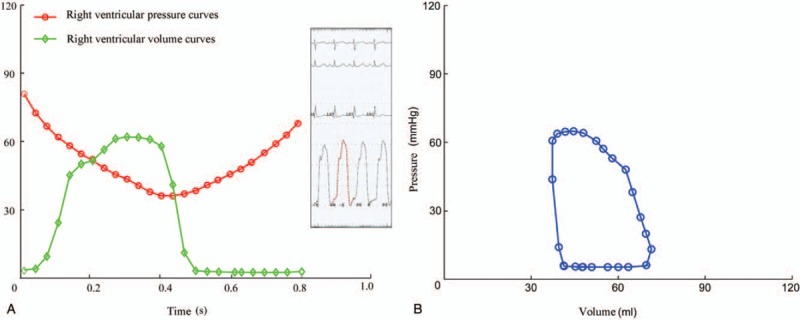
(A and B) Diagram of pressure–volume loop.

At present, the coupling efficiency coefficient (*η*_vv_) was calculated by the pressure–volume loop method.^[5]^ In general, it was calculated using the transient ventricular pressure–volume curve in a specific cardiac cycle. The right ventricular pressure and volume curves were obtained according to the RHC and post-processing of cardiovascular magnetic resonance images. The GetData Graph Digitizer software was used to calibrate time, pressure, and volume, and to digitize pressure and volume curves. Then the MATLAB software was used to draw the pressure and volume curves in the same cardiac cycle (Fig. 1A), and the corresponding pressure–volume loop was produced (Fig. 1B).

**Figure 2:**
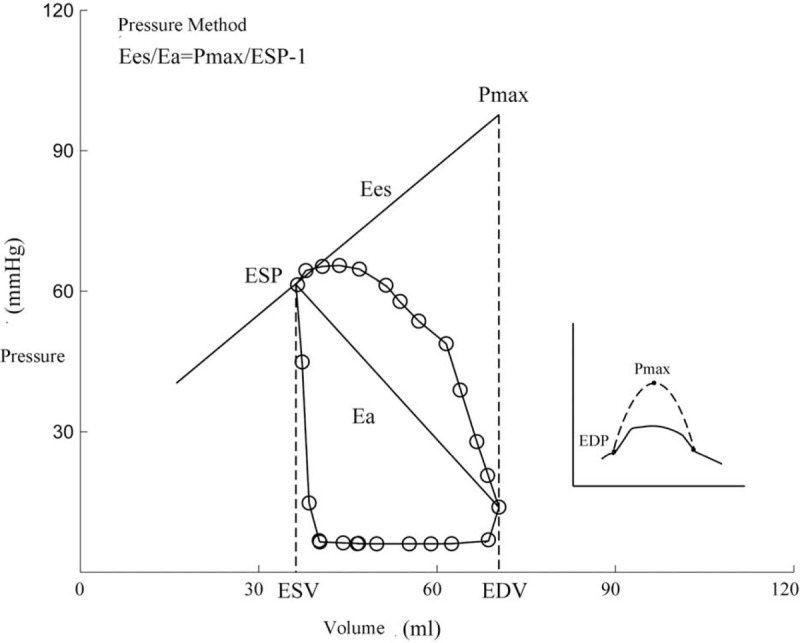
Diagram of the pressure method.

According to pressure–volume loop, 2 main methods were used to calculate the right ventricular–pulmonary coupling efficiency coefficient: pressure method and volumetric method.^[6]^ The pressure method was also known as the single heartbeat method, which was first proposed by Takeuchi et al.^[7]^ Theoretically, *E*es was the slope of the maximum constant-volume pressure (*P*max) to the end-systolic point, that is, (*P*max – ESP)/SV (Fig. 2). The pressure wave of isovolumic contraction was approximate to the sine wave. Therefore, according to the right ventricular pressure waveform diagram, it could be obtained using sine curve fitting, and *P*max was estimated to be the peak value of sine wave. *E*a was the slope of end-systolic point on the pressure–volume loop to the end-diastolic point. Assuming that the right ventricular end-diastolic pressure was 0, arterial elastance (*E*a) could be estimated as ESP/SV. Therefore, coupling efficiency coefficient (*η*_vv_) was calculated using the ratio of *E*es to *E*a. 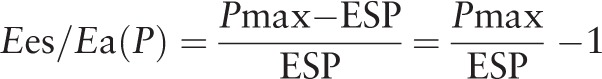


**Figure 3:**
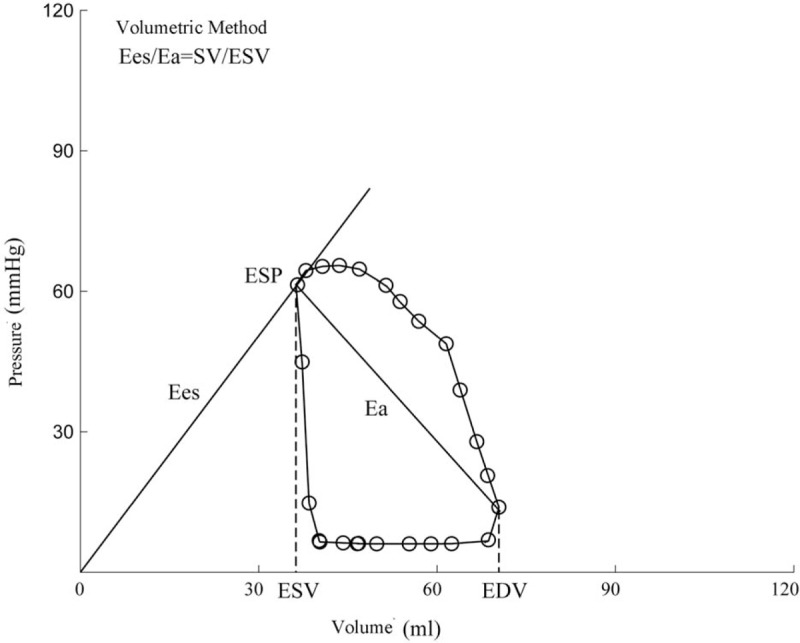
Diagram of the volumetric method.

In addition, the completely noninvasive method was also adopted for calculation. The volumetric method was proposed by Sanz's,^[8]^ of which, *E*es = ESP/ESV (Fig. 3). *E*a was defined as mentioned earlier: 
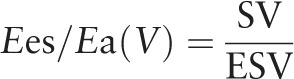


### 2.3 Statistical analysis

Statistical analyses were conducted using SPSS version 22.0 statistical software. The continuous values were tested for normal distribution and expressed as mean ± standard deviation or median (range); the differences between groups were analyzed by a Student *t* test or Mann–Whitney test. Categorical variables are expressed as a proportion, and differences were tested by *χ*^*2*^ analyses. The association between right ventricular–pulmonary artery coupling and the prognosis of patients with PAH was determined by the Spearman correlation analysis. The survival analysis methods included Kaplan–Meier plots, Log-rank test, and Cox modeling. For all analyses, a *P* < .05 was considered as statistically significant.

## 3 Results

### 3.1 Basic information of patients

A total of 64 patients were included in this study, of which 49 (35 females and 14 males; average age 31 ± 13 [7–65] years) were diagnosed with PAH by RHC. Eleven had congenital heart disease–associated PAH (8 with Eisenmenger's syndrome and 3 with delayed pulmonary hypertension after congenital heart disease), 17 had idiopathic PAH (had excluded other etiology), 14 had connective tissue disease-associated PAH, 1 had chronic thromboembolic pulmonary hypertension, and 6 had other PAH. Further, 32 had the World Health Organization (WHO) cardiac function grades I–II and 17 had grades III–IV. The 6-minutes walking distance was 400 ± 120 (0–552) miles. Also, 3 with WHO cardiac function grade IV were unable to walk; the 6-minutes walking distance was 0. The drug therapy for PAH included 21 patients taking phosphodiesterase type 5 inhibitors, 18 taking endothelin receptor antagonists, 2 taking prostaglandin, 31 taking warfarin, 17 taking diuretics, and 17 taking digitalis.

Fifteen patients (10 females and 5 males; average age 38 ± 14 [9–57] years) in the control group (non-PAH) were treated with patent foramen ovale occlusion. Eleven had WHO cardiac function grades I–II, 3 had grade III, and no patient had grade IV; the 6-minutes walking distance was 443 ± 93 (170–550) m. No significant difference in age, gender, WHO cardiac function classification, and 6-minutes walking distance was found between the 2 groups (Table 1).

**Table 1:**
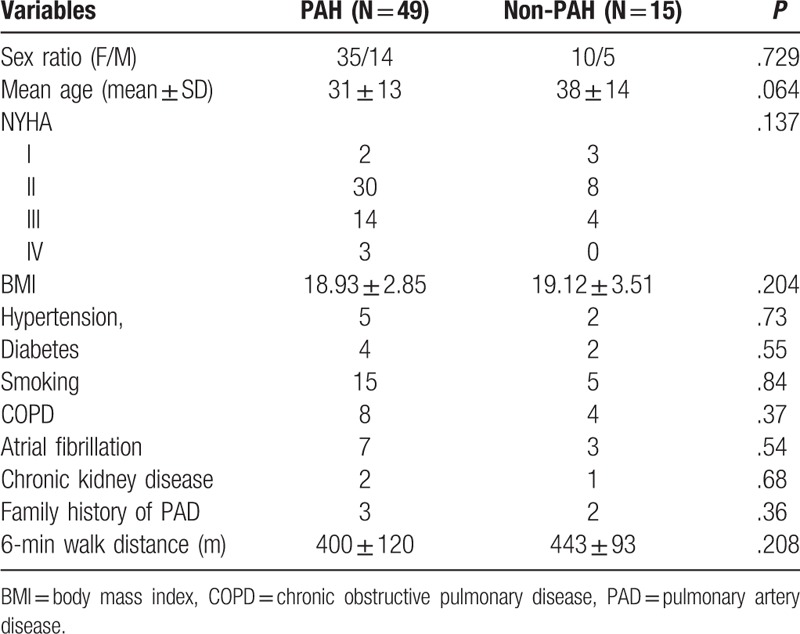
Clinical characteristic of patients.

The data of hemodynamic parameters, right ventricular volume parameters, and right ventricular–pulmonary artery coupling are shown in Table 2. Hemodynamic parameters, including mPAP, RVESP, RVEDP, and PVR, of patients with PAH were significantly higher than those of patients with non-PAH, and the differences were statistically significant (*P* < .05). For right ventricular volume parameters, the RVESV, RVEDV, and SV of patients with PAH were much higher than those of patients with non-PAH patients. Their RVEF was less than that of patients with non-PAH, and the differences were statistically significant (*P* < .05). For right ventricular–pulmonary artery coupling parameters, no difference was observed between the 2 groups, irrespective of the method used (*P* > .05). The *E*a of patients with PAH was about twice that of patients with non-PAH. The *E*es/*E*a was less than that of patients with non-PAH, and the differences were statistically significant (*P* < .05). In addition, *E*es/*E*a calculated by the pressure method was obviously smaller than that calculated by the volumetric method.

**Table 2:**
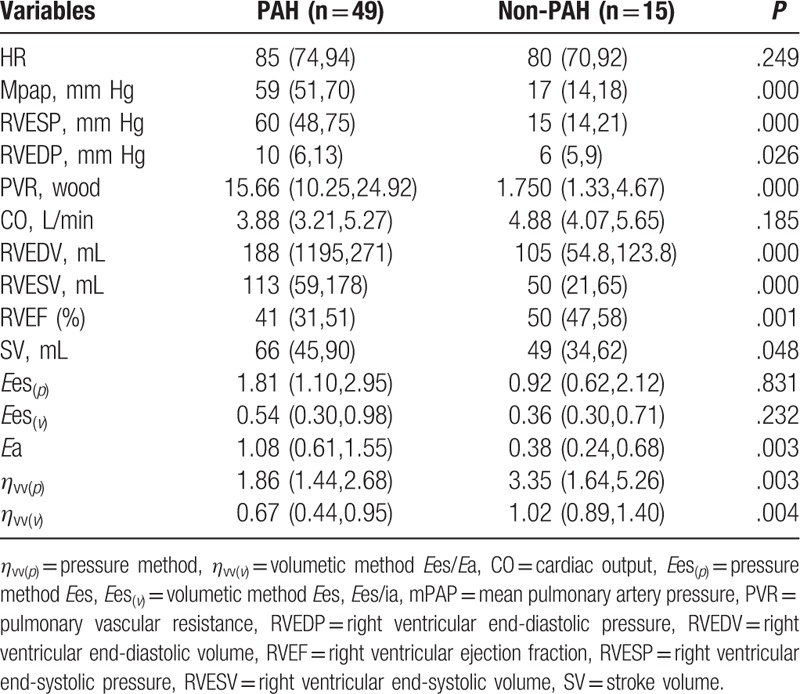
The data of hemodynamic parameters, right ventricular volume parameters, and right ventricular–pulmonary artery coupling.

In the PAH group, the correlations of right ventricular–pulmonary artery coupling with baseline hemodynamic parameters and right ventricular volume parameters are shown Table 3.

**Table 3:**
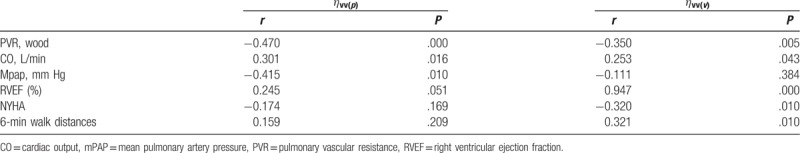
The correlations of right ventricular–pulmonary artery coupling with baseline hemodynamic parameters and right ventricular volume parameters.

The results showed a significant negative correlation of PVR with *η*_vv(*p*)_ and *η*_vv(*v*)_ (*P* < .05); that is, the greater the PVR, the smaller the *E*es/*E*a. CO was significantly and positively correlated with *η*_vv(*p*)_ and *η*_vv(*v*)_ (*P* < .05); that is, the greater the CO, the greater the *E*es/*E*a. Moreover, *η*_vv(*p*)_ was only negatively correlated with mPAP (*r* = –0.415; *P* < .05); that is, the greater the average pulmonary artery pressure, the smaller the *η*_vv(*p*)_. *η*_vv(*v*)_ was significantly and positively correlated with RVEF (*r* = 0.947, *P* < .01) and 6-minutes walking distance (*r* = 0.321, *P* < .05). It was significantly and negatively correlated with cardiac function classification (*r* = –0.320, *P* < .05); that is, the higher the right ventricular ejection fraction, the farther the 6-minutes walking distance, the lower the cardiac function classification, and the greater the *η*_vv(*v*)_. At the same time, a significant positive correlation was observed between *η*_vv(*p*)_ and *η*_vv(*v*)_ (*r* = 0.294, *P* < .05).

### 3.2 Analysis of factors influencing survival time

Hemodynamic parameters, right ventricular volume parameters, and right ventricular–pulmonary artery coupling parameters were incorporated into the univariate Cox regression model. Forward logistic regression (maximum-likelihood estimation) was used for analysis. The log-likelihood ratio test was used (*χ*^2^ = 4.721, *P* = .030), indicating statistical significance in the test of overall model. The variable included in the equation was *η*_vv(*v*)_ (odds ratio = 0.192, 95% confidence interval: 0.042–0.868, *P* = .032), indicating that *E*es/*E*a calculated by the volumetric method was an independent index to predict survival time.

### 3.3 Analysis of survival time

**Figure 4:**
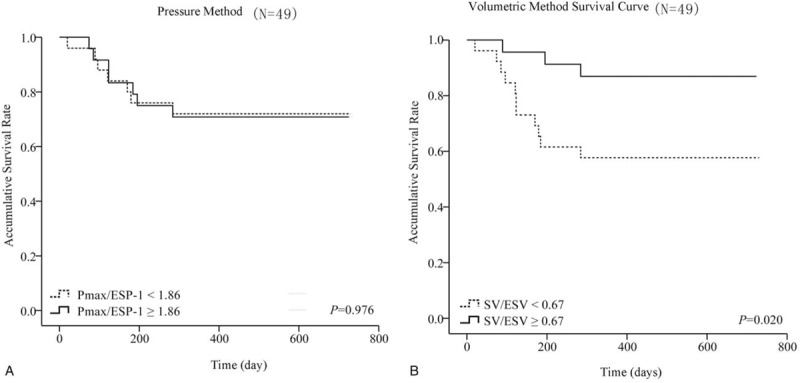
(A) The survival rate of pressure method; (B) The survival rate of volumetric method.

The follow-up period was 2 years, with a total of 14 endpoint events; 3 patients died, and 11 clinically deteriorated. *η*_vv(*p*)_ and *η*_vv(*v*)_ were grouped according to the median, and the difference in survival time between groups was compared. For patients with PAH (N = 49), grouping was conducted according to *η*_vv(*p*)_ = 1.86 and *η*_vv(*v*)_ = 0.67. No difference in survival time was recorded between 2 groups of *E*es/*E*a calculated by the pressure method (log-rank test value = 0.001, *P* = .976) (Fig. 4A). However, a significant difference existed in survival time between the 2 groups of *E*es/*E*a calculated by the volumetric method (log-rank test value = 5.398, *P* = .020) (Fig. 4B).

## 4 Discussion

The survival time of patients with PAH has been comprehensively evaluated and analyzed using multiple factors, including hemodynamic data, clinical functional indexes, ultrasound indexes, biochemical indexes, nuclear magnetic resonance, and other indexes. These indexes have their own advantages and disadvantages. At present, the consensus was that a single index could not be used to evaluate the prognosis of patients with PAH. Therefore, a comprehensive evaluation of multiple parameters was needed.^[9]^ Moreover, a great difference existed in the evaluation indexes and measuring methods selected at different PAH centers, leading to the perplexity and uncertainty among clinicians on the long-term prognosis of these patients. Therefore, finding more valuable prognostic parameters should be the focus of clinicians and researchers in the future. Right cardiac catheterization is the gold standard to evaluate hemodynamic indexes in patients with PAH. Also, cardiac MRI is the most accurate method to evaluate right ventricular volume parameters. The combination of the 2 forms the right ventricular–pulmonary artery coupling coefficient to predict the long-term survival time of patients with PAH, which was the main purpose of this study.

Two methods are used to calculate the efficiency of right ventricular–pulmonary artery coupling based on the pressure–volume loop: pressure method and volumetric method. The *η*_vv(*p*)_ values obtained from the comprehensive pressure and volume curves, and the *η*_vv(*v*)_ obtained from the volume curve only, were compared with those in the normal control group, that is, patients with non-PAH. Burkhoff revealed that irrespective of the method used_,_*η*_vv_ was significantly different from that in patients with PAH (*P* < .01), indicating that *η*_vv_ was a new index and its baseline significance was consistent with that of common clinical indexes, such as mPAP, RVESP, RVEDP, RVEDV, RVESV, RVEF, and RVSV. However, no statistically significant difference in *E*es was found, reflecting right ventricular elastance calculated by the 2 methods in patients with PAH and non-PAH, which was consistent with CO and 6-minutes walking distance (no statistical difference was found in the normal control group). It was believed that this phenomenon was related to the cardiac function status of selected patients. Considering the compliance of MRI examination, 94% patients with PAH had WHO cardiac function grade above III. With the gradual increase in pulmonary arterial pressure or PVR, the right ventricle of patients with PAH must be enlarged to adapt to the increased after-loading. The increased right ventricular systolic function led to a progressive disorder. ESP and EDP increased. Before decompensation, CO, 6-min walk distance, and *E*es, which were used to evaluate the contractile force of the right ventricle, were basically normal, resulting in no statistical difference in *E*es at baseline. Takeuchi et al reported ESV and EDV increased significantly with the further enlargement of the right ventricle. When self-regulation could not maintain appropriate SV, the right ventricular function began to collapse; *E*es, CO, and 6-minute walk distance decreased at that time. Further, *E*a, as an index of effective PA elastance, increased abnormally at baseline (*P* < .01), indicating that the initial stage of increased pulmonary arterial pressure in patients with PAH, that is, the changes in PA elastance and contractility, was consistent with mPAP and PVR. However, whether the long-term change rate could reflect prognosis needed further investigation.

For patients with PAH, a further correlation analysis showed a significant negative correlation between *η*_vv(*p*)_ and *η*_vv(*v*)_ with PVR at baseline (*P* < .05). However, they significantly and positively correlated with CO (*P* < .05), which was coincident with Kuehne's report. That is, the greater the PVR, the smaller the CO, and the smaller the *E*es/*E*a of the right ventricular–pulmonary artery coupling coefficient. Also, *η*_vv_ could be used to comprehensively evaluate the myocardial contractility and the resistance of pulmonary vascular bed in patients with PAH. Further analysis showed that only *η*_vv(*p*)_ in the pressure method was negatively correlated with mPAP (*r* = –0.415, *P* < .05). That is, the greater the average pulmonary arterial pressure, the smaller the *η*_vv(*p*)_. However, the *η*_vv(*v*)_ in the volumetric method significantly and positively correlated with RVEF (*r* = 0.947, *P* < .01) and 6-minutes walking distance (*r* = 0.321, *P* < .05), and it significantly and negatively correlated with cardiac function classification (*r* = –0.320, *P* < .05. That is, the higher the right ventricular ejection fraction, the farther the 6-minutes walking distance, the lower the cardiac function grade, and the greater the *η*_vv(*v*)_. Previous animal experiments and clinical studies on the evaluation of the efficiency of the right ventricular–pulmonary artery coupling showed that the coupling efficiency of healthy state and diseased state was significant. The coupling efficiency of the healthy state was larger, and the coupling efficiency of the disease state was lower.^[10,11,12]^ The results of this study also confirmed the conclusion that for patients with higher mPAP and lower CO, the lower the *η*_vv(*p*)_, the worse their health status; for patients with lower heart function grade, the higher the EF and the longer the 6-minutes walking distance, the higher the η_vv(*v*)_, and the better their health status. These results further indicated that the *η*_vv_ measured by 2 different methods reflected the basic state of patients with PAH at baseline.

Among the aforementioned indexes, CO, PVR, 6-minutes walking distance, and WHO cardiac function classification were clearly instructive for the clinical prognosis of patients with PAH. Further, *η*_vv(*v*)_ clearly correlated with these indexes, and *η*_vv(*p*)_ only correlated with some of the indexes, explaining the different values obtained using the 2 methods in clinical prognosis. It was interesting that although the right ventricular–pulmonary artery coupling coefficient (*η*_vv_) was a new index used to evaluate the long-term prognosis of patients in clinical practice,^[13]^ the parameters (*η*_vv(*p*)_ and *η*_vv(*v*)_) measured by the 2 methods also significantly and positively correlated with each other (*r* = 0.294, *P* < .05), indicating that the introduction of different indexes using different computational methods resulted in great differences. Therefore, the standardization and calculation of *E*es/*E*a, besides the correct evaluation of the long-term prognostic value of the aforementioned indexes, were directly related to the clinical application and significance of *E*es/*E*a.

After 2-year follow-up with hard endpoints, 14 endpoint events (3 patients died) were observed. Further, *η*_vv(*p*)_ and *η*_vv(*v*)_ were grouped according to medians, and the differences in survival time between groups were compared. The analysis of the factors influencing the survival time showed no significant difference in *η*_vv(*p*)_ measured by the pressure method between groups (log-rank test value = 0.001, *P* = .976), whereas the efficiency coefficient of coupling (*η*_vv(*v*)_) measured by the volumetric method was an independent predictor (log-rank test value = 5.398, *P* = .020). While analyzing the survival time, the *η*_vv(*v*)_ of patients with PAH was divided into 2 groups according to the median of 0.67. No statistically significant difference was found between groups, indicating that SV/ESV = 0.67 could be used as a critical value for the deterioration of patients with PAH. This was basically the same as the result of Rebecca et al^[6]^ that SV/ESV = 0.515 was the critical value to predict the survival time of patients with PAH. However, the *η*_vv(*p*)_ calculated by the more complex pressure method could not reflect clinical prognosis. This result was of great significance for patients with PAH: the simple right ventricular–pulmonary coupling coefficient derived from noninvasive cardiac MRI was superior to the value obtained using invasive RHC.

## 5 Limitations

The major limitation of this study is the small sample, which was not from multicenter and different race. The follow-up time was not long enough, which may reduce the persuasion of this study. We could not completely exclude the population from other risk factors that affect survival statue. The absolute correlation between right ventricular–pulmonary artery coupling and the prognosis of patients with PAH in long term still need to be identified furthermore.

## 6 Future directions

In this study, it was assumed that the diagnosis and treatment of patients with PAH could be improved as follows: for the first diagnosis, the right cardiac catheterization and other invasive examinations were needed to confirm the diagnosis of PAH. After 6 months to 1 year, the right cardiac catheterization could be replaced by nuclear magnetic resonance. In recent years, ventricular–vascular coupling indexes were constantly accuralized or even redefined in terms of the prominent features of the right ventricle. The use of noninvasive methods to measure the right ventricular function was expected to monitor the development of the disease and helped to select specific treatment options for patients.

## 7 Conclusions

PAH is a complex disease, which has greatly threatened human health and life. It is urgent to correctly evaluate and predict the prognosis of the patients with PAH. The right ventricular–pulmonary artery coupling (*η*_vv_) is important to define the cardiac function in PAH. The *E*es/*E*a calculated by the volumetric method may be better to predict the prognosis of PAH. When *E*es/*E*a <0.67, the patients with PAH were more likely to turn to a rapid deterioration.

## Author contributions

**Data curation:** Sanping Zhang.

**Formal analysis:** Yaling Dong.

**Investigation:** Menghuan Yan.

**Methodology:** Jun Li.

**Resources:** Ming Xu.

**Software:** Lin Nie.

**Validation:** Gangcheng Zhang.

**Writing – original draft:** Laichun Song.

**Writing – review & editing:** Jun Li, Laichun Song.

## References

[1] Bellofiore A Chesler NC Methods for measuring right ventricular function and hemodynamic coupling with the pulmonary vasculature. Ann Biomed Eng 2013;41:1384–98. 23423705 10.1007/s10439-013-0752-3 PMC3679286

[2] Burkhoff D Sagawa K Ventricular efficiency predicted by an analytical model. Am J Physiol 1986;250:1021–7. 10.1152/ajpregu.1986.250.6.R1021 3717375

[3] Abel FL Fourier analysis of left ventricular performance. Evaluation of impedance matching. Circ Res 1971;28:119–35. 4994209 10.1161/01.res.28.2.119

[4] Galiè N Humbert M Vachiery JL 2015 ESC/ERS guidelines for the diagnosis and treatment of pulmonary hypertension. Kardiologia Polska 2015;73:1127. 26727670 10.5603/KP.2015.0242

[5] Chirinos JA Ventricular-arterial coupling: invasive and non-invasive assessment. Artery Res 2013;7:2–14. 10.1016/j.artres.2012.12.002 PMC3809068 24179554

[6] Vanderpool RR Pinsky MR Naeije R RV-pulmonary arterial coupling predicts outcome in patients referred for pulmonary hypertension. Heart 2015;101:37–43. 25214501 10.1136/heartjnl-2014-306142 PMC4268056

[7] Takeuchi M Igarashi Y Tomimoto S Single-beat estimation of the slope of the end-systolic pressure-volume relation in the human left ventricle. Circulation 1991;83:202–12. 1898642 10.1161/01.cir.83.1.202

[8] Trip P Kind T Van de Veerdonk MC Accurate assessment of load-independent right ventricular systolic function in patients with pulmonary hypertension. J Heart Lung Transplant 2013;32:50–5. 23164535 10.1016/j.healun.2012.09.022

[9] Burkhoff D Mirsky I Suga H Assessment of systolic and diastolic ventricular properties via pressure-volume analysis: a guide for clinical, translational, and basic researchers. Am J Physiol Heart Circ Physiol 2005;289:H501–12. 16014610 10.1152/ajpheart.00138.2005

[10] Fourie PR Coetzee AR Bolliger CT Pulmonary artery compliance: its role in right ventricular-arterial coupling. Cardiovasc Res 1992;26:839–44. 1451160 10.1093/cvr/26.9.839

[11] Kuehne T Yilmaz S Steendijk P Magnetic resonance imaging analysis of right ventricular pressure-volume loops: in vivo validation and clinical application in patients with pulmonary hypertension. Circulation 2004;110:2010–6. 15451801 10.1161/01.CIR.0000143138.02493.DD

[12] Sanz J Garcíaalvarez A Fernándezfriera L Right ventriculo-arterial coupling in pulmonary hypertension: a magnetic resonance study. Heart 2012;98:238–43. 21917658 10.1136/heartjnl-2011-300462

[13] Brewis MJ Bellofiore A Vanderpool RR Imaging right ventricular function to predict outcome in pulmonary arterial hypertension. Int J Cardiol 2016;218:206–11. 27236116 10.1016/j.ijcard.2016.05.015 PMC5001160

